# Sex Differences in Long-Term Survival and Cancer Incidence After Ruptured Abdominal Aortic Aneurysm Repair

**DOI:** 10.3390/jcm13226934

**Published:** 2024-11-18

**Authors:** Jasmin Epple, Dittmar Böckler, Reinhart T. Grundmann

**Affiliations:** 1 Department of Vascular and Endovascular Surgery, University Hospital Heidelberg, 69120 Heidelberg, Germany; 2 University Heart and Vascular Center Hamburg, Department for Vascular Medicine, University Hospital Hamburg-Eppendorf, 20251 Hamburg, Germany

**Keywords:** ruptured abdominal aortic aneurysm, cancer incidence, gender, long-term outcome, octogenarians

## Abstract

**Background:** Long-term gender-specific survival and cancer incidence in patients with ruptured abdominal aortic aneurysm (rAAA) were investigated after endovascular (EVAR) and open repair (OAR). **Methods**: Data from 2933 patients (EVAR n = 1187, OAR n = 1746) from a health insurance company in Germany (men n = 2391, women n = 542) were analyzed. All patients were cancer-free in their history. **Results**: Perioperative mortality was significantly higher after OAR (42.6%) than after EVAR (21.2%; *p* < 0.001). Women had significantly higher in-hospital mortality (41.5%) than men (32.2%). Notably, the 5-year survival was 36.9% after OAR and 40.8% after EVAR (*p* < 0.001), and 40.7% in men and 29.1% in women (*p* < 0.001). Overall, 17.2% of EVAR and 14.6% of OAR patients had cancer at 5 years (*p* = 0.328). Cancer incidence did not differ significantly between men and women. Patients with cancer had a significantly less favorable outcome compared to patients with no cancer (*p* = 0.002). Treatment of rAAA was also indicated in octogenarians, with survival rates of 19.9% after 5 years and even 38.4% with perioperative deaths excluded. **Conclusions**: Cancer represents a significant risk factor for survival in patients with rAAA. These patients should be monitored during follow-up, particularly regarding the development of lung cancer.

## 1. Introduction

This study focuses on the long-term gender-specific survival of patients with ruptured abdominal aortic aneurysm (rAAA) following endovascular (EVAR) and open repair (OAR). Our aim is to determine the expected tumor incidence during follow-up and to specifically assess the survival of patients over 80 years old with rAAA, for which relatively few data are available. In the randomized IMPROVE trial [1], after more than 3 years of follow-up, 179/316 (56.6%) deaths were observed following rAAA repair with EVAR, including 19/179 (10.6%) due to cancer. In the OAR group, there were 183/297 (61.6%) deaths, of which 13/183 (7.1%) were cancer-related. Specific gender-related data were not provided. Varkevisser et al. [2] reported a 5-year survival rate of 63% after EVAR and 52% after OAR for rAAA repair in a cohort from the SVS Vascular Quality Initiative (VQI) clinical registry (2013–2019), but did not provide information on tumor incidence or patient gender. Ettengruber et al. [3] examined a cohort of patients with intact abdominal aortic aneurysm (iAAA). They found that patients with cancer had a significantly worse outcome compared to those without cancer (HR 1.68; 95% CI 1.59–1.78, *p* < 0.001). After nine years, the estimated survival rates were 27.0% for patients with cancer and 55.4% for those without (*p* < 0.001). A comparable study on rAAA does not yet exist. Li et al. [4] reported a survival rate of 36.7% for women vs. 49.5% for men (*p* = 0.02) after endovascular and open AAA repair in a multicenter retrospective cohort study using prospectively collected VQI data, including 1160 women and 4148 men with rAAA, followed up for up to 8 years. Women had higher perioperative and long-term mortality. Data from the Dutch Surgical Aneurysm Audit (DSAA) were analyzed by Alberga et al. [5], including 2879 patients, of whom 1146 were treated with EVAR (382 octogenarians, 33%) and 1733 with OAR (410 octogenarians, 24%). The perioperative mortality rate for all octogenarians was 43.8% compared with 24.1% in all non-octogenarians. Although aneurysm repair was associated with high mortality in this patient category, especially after OAR, these authors noted that a substantial proportion of octogenarians (1/3 after EVAR and 1/5 after OAR) experienced an uneventful recovery after rAAA repair. Roosendaal et al. [6] reported a 2-year mortality rate of 66% (25/38) after EVAR and 62% (34/55) after OAR in 110 octogenarian patients with rAAA. They concluded that surgery for rAAA in active octogenarians should not be denied based on age alone. Gender-specific data or cancer incidence were not reported. Also, given the poor prognosis for patients undergoing rAAA repair, it remains uncertain whether a tumor would still play a significant role in long-term survival. This study aims to fill this gap by presenting long-term outcomes and cancer incidence after rAAA repair in men and women, as well as in patients under and over 80 years old, based on a large database from a German health insurance provider.

## 2. Materials and Methods

In this retrospective study, patient data from the nationwide health insurance company, AOK-Die Gesundheitskasse, were analyzed. The data were provided and anonymized by WIDO (Wissenschaftliches Institut der AOK). The analysis of the health insurance data involved an evaluation of the complete medical history of each patient. Therefore, this study is not limited to a specific hospital group or medical department. All diagnoses and procedures were coded using the International Classification of Diseases, Tenth Revision (ICD-10) and Operations and Procedures (OPS) codes. 

This study includes data on all patients who underwent either endovascular (OPS code: 5-38a.1) or open repair (OPS code: 5-384.7) of a ruptured abdominal aortic aneurysm (rAAA) (ICD code: I71.3 abdominal aortic aneurysm, ruptured) between 1 January 2010 and 31 December 2016. A total of 3227 patients with rAAA were identified. A total of 294 patients with a history of cancer were excluded from the study. Cancer was identified if a cancer-specific ICD was found in the insurance database prior to aneurysm repair (ICD codes: C00-C97). The remaining 2933 rAAA patients were then divided into two groups according to gender. An analysis of comorbidities and perioperative and postoperative complications was performed using documented ICD and OPS codes. The AOK dataset is comprehensive, capturing standardized billing and diagnostic information from all healthcare facilities. As a result, missing data in this dataset are minimal and generally limited to clinical information not covered by ICD or OPS coding, such as specific lifestyle factors or laboratory results. Where such information was unavailable, no imputation was performed; analyses were strictly based on the coded data available. Since it was not possible to determine whether the deaths occurred in the treating hospital, after transfer, or during rehabilitation, perioperative mortality was defined as 60-day mortality. All patients were followed up until 31 December 2018. 

### Statistical Analysis

The analysis was performed with SPSS 27 (IBM Deutschland GmbH, Ehningen, Germany). To assess significant differences between the two genders, Chi-square tests were performed for non-metric variables. For metric variables, a Mann–Whitney U-test was used. 

For estimating overall survival and cancer incidence in the follow-up, Kaplan–Meier tables were generated, and the log-rank test was used to test for significance between the two groups. A univariable Cox proportional model was implemented before a multivariable Cox proportional model to assess the impact of comorbidities, age, and gender on overall survival. All parameters demonstrating a statistically significant influence on survival in the univariable analysis (*p* < 0.05) were tested in the multivariable analysis. Significance was assessed using log-rank tests. *p*-values less than 0.05 were considered statistically significant.

## 3. Results

### 3.1. Patients

Patient characteristics and comorbidities are shown in Table 1. In the rAAA cohort, 2391 patients were male (81.5%), and 542 were female (18.5%). A total of 40.8% of male patients underwent EVAR compared to 38.9% of female patients (*p* = 0.418). A total of 59.2% of male patients and 61.1% of female patients underwent OAR (*p* = 0.418). Women were significantly older than men with a mean age of 79.6 vs. 73.9 years (*p* < 0.001). Female patients had significantly more diabetes mellitus type 2 (male: 10.8%, female: 15.9%, *p* = 0.001) and arterial hypertension (male: 35.1%, female: 47.4%, *p* < 0.001). A history of myocardial infarction was significantly more common in men (men: 8.4%, women: 5.2%, *p* = 0.011).

### 3.2. Perioperative Outcome

Perioperative outcomes are shown in Table 2. Perioperative mortality was 32.2% for men and 41.5% for women (*p* < 0.001). In EVAR patients, 20.6% of men and 24.2% of women died perioperatively (*p* = 0.249). For OAR, the mortality rates were 40.2% for men and 52.6% for women (*p* < 0.001). Men aged ≥ 80 years had a mortality rate of 47.6% (EVAR 32.7%, OAR 60.6%) compared to 50.3% (EVAR 27.0%, OAR: 65.1%) for women (*p* = 0.416). In comparison, perioperative mortality in patients < 80 years was lower with 25.6% (EVAR: 14.2%, OAR: 32.7%) in men and 29.4% (EVAR: 20.2%, OAR: 35.3%) in women (*p* = 0.222).

Hospital stay did differ significantly between male (mean: 26.6 days) and female patients (mean: 30.3 days, *p* = 0.002) and was longer in patients ≥ 80 years (mean: 28.0 days) compared to patients < 80 years (mean: 26.9 days, *p* = 0.040) if the patients survived the operation. 

Female patients were significantly more likely to require blood transfusions (83.4%) than male patients (77.0%) (*p* = 0.001). All other perioperative complications analyzed did not differ significantly between the two genders.

### 3.3. Long-Term Survival

**Figure 1:**
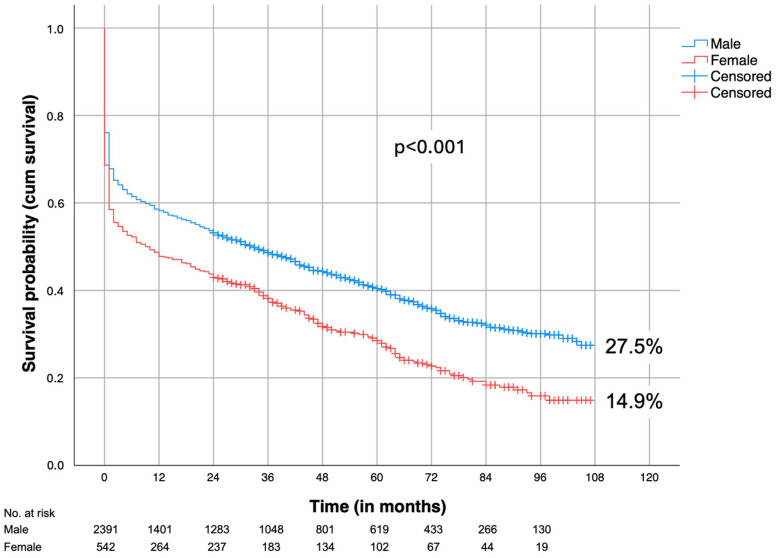
Survival (all patients were cancer-free at time of repair); male vs. female.

The 5-year survival of male and female patients, differentiated by age and EVAR and OAR, is given in Table 3. In total, 40.8% of EVAR and 36.9% of OAR patients survived 5 years (*p* < 0.001). A total of 40.7% of male patients were still alive compared to 29.1% of female patients (*p* < 0.001, Figure 1). If the patients survived the initial repair (perioperative deaths excluded), 60.1% of male and 49.7% of female patients were still living after 5 years (*p* < 0.001).

**Figure 2:**
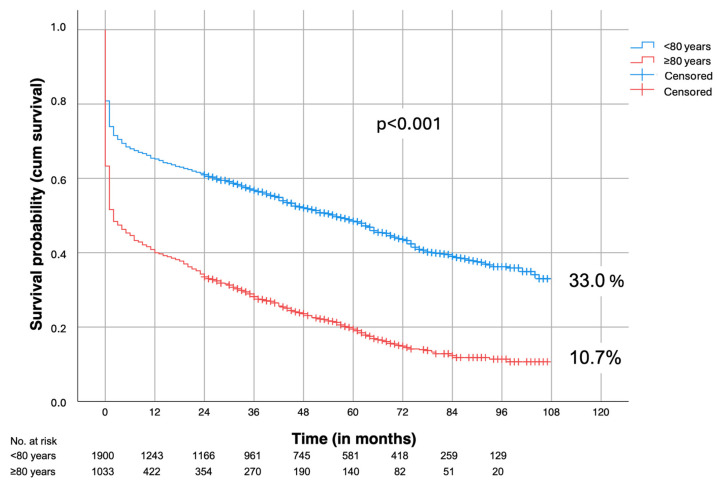
Survival (all patients were cancer-free at time of repair); <80 years vs. ≥80 years.

A total of 48.8% of patients < 80 years and 19.9% of patients ≥ 80 years survived 5 years (*p* < 0.001, Figure 2).

The factors influencing long-term outcomes were identified for patients who survived the initial rAAA repair (Table 4). Age ≥ 80 years (HR: 2.013, *p* < 0.001), COPD (HR: 1.602, *p* < 0.001), chronic kidney disease stage 3–5 (HR: 1.545, *p* < 0.001), cancer (HR: 1.318, *p* = 0.002), and left heart failure (NYHA 2–4) (HR: 1.269, *p* = 0.043) negatively influenced survival.

Gender had no significant effect on survival (HR: 1.161, *p* = 0.084).

### 3.4. Cancer Incidence in the Follow-Up

In total, 17.2% of EVAR patients and 14.6% of OAR patients were diagnosed with cancer after five years (*p* = 0.328). Cancer incidence was not significantly higher in men (16.5%) compared to women (11.9%) (*p* = 0.153) (Table 5). This pattern was observed for both EVAR (men: 18.2%, women: 11.0%, *p* = 0.272) and OAR (men: 15.2%, women: 12.1%, *p* = 0.305). Lung cancer was the most common cancer during follow-up, affecting 4.4% of men and 2.7% of women (*p* = 0.288). The incidence of ureter and bladder cancer at five years (men: 2.8%, women: 1.0%, *p* = 0.098) and colon cancer (men: 2.0%, women: 1.4%, *p* = 0.598) did not differ significantly between the sexes. Prostate cancer affected 2.5% of men after five years of follow-up.

**Figure 3:**
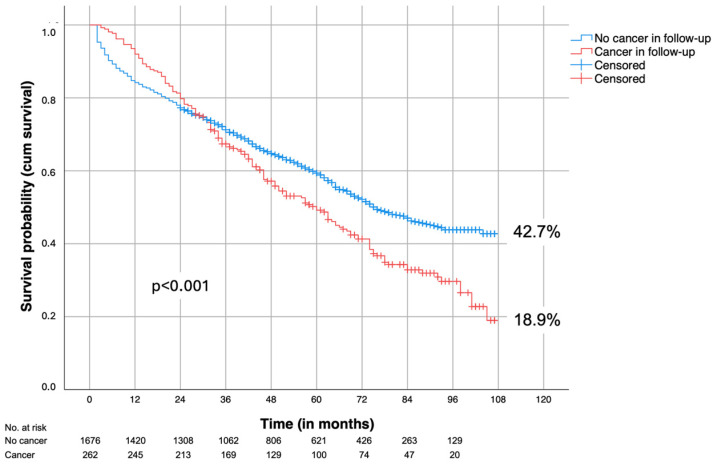
Survival of cancer-free patients and patients with cancer in the follow-up (all patients were cancer-free at time of repair. Perioperative deaths excluded).

Figure 3 shows the post-discharge survival of patients who survived rAAA repair (excluding in-hospital mortality), differentiated by whether they developed cancer during follow-up. After five years, 59.9% of cancer-free patients and 50.5% of cancer patients were still alive (*p* < 0.001).

## 4. Discussion

In our study, in-hospital mortality following rAAA repair was significantly higher after OAR at 42.6% compared to 21.2% after EVAR (*p* < 0.001). This finding aligns with the results of a comprehensive literature review and a meta-analysis of approximately 267,259 patients from 136 studies, which demonstrated that EVAR carries a lower perioperative mortality risk than open surgery (Kontopodis et al. [7]). Women had a significantly higher in-hospital mortality rate of 41.5% following rAAA repair compared to 32.2% in men, which was attributable solely to the higher mortality rate associated with OAR, as no significant gender differences were observed after EVAR (Table 2). Similarly, in the IMPROVE trial [8], women particularly benefited from the endovascular approach, with a 30-day mortality rate of 37% after EVAR versus 57% after OAR. A higher in-hospital mortality rate was also reported by Ho et al. [9] in the Vascular Quality Initiative (VQI) registry from 2013 to 2019 for rAAA patients. In this study, which included a total of 1775 patients (23.8% female), the in-hospital mortality rate after EVAR was 45.9% in women compared to 34.5% in men (*p* < 0.01).

In this study, the 5-year survival rate was 36.9% after OAR and 40.8% after EVAR (*p* < 0.001), which is less favorable than the survival rates reported by Varkevisser et al. [2] (63% after EVAR and 52% after OAR). However, Varkevisser et al. did not specify the percentage of patients over 80 years old, which in our study was 30.1% for men and 57.9% for women. Among patients who survived the initial AAA repair (excluding in-hospital mortality), better post-discharge survival was observed after OAR compared to EVAR, with 64.2% vs. 51.8% at 5 years (*p* < 0.001). This was also confirmed by multivariate regression analysis (HR: 0.838 [CI, 0.731–0.960]; *p* = 0.011).

In contrast, Salata et al. [10] found in a population-based study of 2692 rAAA patients (261 EVAR [10%] and 2431 OAR [90%]) that there were lower hazards for all-cause mortality and major adverse cardiovascular events (MACEs) within 30 days of surgery in favor of EVAR but no differences in mid- or long-term outcomes. Similarly, Kontopodis et al. [11] reported in a meta-analysis involving a total of 31,383 patients that there were no significant differences in the hazard of death after discharge from the hospital between the EVAR and open repair groups (HR, 1.10; 95% CI, 0.85–1.43; *p* = 0.47).

In our study, men exhibited a significantly better 5-year survival rate of 40.7% compared to 29.1% in women (*p* < 0.001) (Figure 1). This trend persisted even when excluding in-hospital mortality, with a 5-year survival rate of 60.1% for men and 49.7% for women, which can be attributed to the higher age of the female patients.

Li et al. [4] also found a less favorable long-term survival for women compared to men in an analysis of the Vascular Quality Initiative database, extending up to 8 years after rAAA repair, and similarly attributed this to the older age of the female patients. However, unlike the study by Li et al., no gender-specific differences were found in the proportion of patients with chronic kidney disease in the current study. The only significant differences observed were a higher prevalence of diabetes and arterial hypertension among women (Table 1).

Biancari et al. [12] reported on 200 patients aged 80 years or older who underwent emergency open repair for rAAA. They found a survival rate of 68% at three years and 45% at five years among the 82 patients who survived the procedure, concluding that an active approach is warranted even in this age group for hemodynamically stable patients. Roosendaal et al. [6] reached a similar conclusion in their analysis of outcomes in 110 octogenarian patients who underwent either endovascular or open repair of rAAA. In their study, half of the patients were still alive one year after the procedure, and more than 80% were living at home, comparable to the general population of 80-year-olds in the Netherlands, where approximately 14% reside in nursing homes. Thus, age alone should not be a criterion to question the feasibility of rAAA repair. This is further demonstrated by the present study, which observed a 5-year survival rate of 19.9% among patients over 80 years old (Figure 2) and an even higher survival when perioperative deaths were excluded (38.4%).

Pirinen et al. [13] analyzed 9464 patients with rAAA repair in Finland and Sweden. The mean survival after EVAR was 7.5 (95% CI 6.9–8.1 years) for patients younger than 65 years, 5.1 (5.4–5.3 years) for patients aged 65–79 years, and 3.1 (2.7–3.4 years) for patients aged >80 years. The results showed that the long-term survival of patients aged 80 years or older after successful AAA treatment is similar to that of the general population. It seems important, however, to consider not only the age but also the frailty of the patient when determining the indication for surgery. Yu et al. [14] identified 5806 patients (age, 72 ± 9 years; 77% male; EVAR, 65%) with rAAA in the VQI database, of whom 36% were frail and 10% were very frail. The overall observed 1-year mortality rate was 52.7%, with higher mortality in the OAR group compared to the EVAR group (55.8% vs. 51.1%; *p* = 0.001). Among very frail patients (n = 566), the 1-year mortality was 70% with EVAR vs. 69.9% with OAR, though patient age was not specifically analyzed. These authors concluded that very frail patients lack the resilience to withstand the stressors of aortic rupture, regardless of the repair technique.

In this study, all patients were cancer-free at the time of repair. After a follow-up period of 5 years, 16.5% of men and 11.9% of women developed cancer (Table 5). Patients who developed cancer had a significantly less favorable outcome compared to those without cancer (HR 1.318; 95% CI 1.112–1.564, *p* = 0.002). Ettengruber et al. [3] analyzed 18,802 iAAA patients. They concluded that patients with a history of cancer had worse long-term survival than those without (HR 1.68; 95% CI 1.59–1.78, *p* < 0.001). At the nine-year mark, the estimated survival rates were 27.0% for patients with cancer and 55.4% for those without (*p* < 0.001). They also analyzed tumor incidence after EVAR and OAR, and EVAR showed an increased risk in postoperative development of abdominal cancer (HR 1.20; 95% CI 1.07–1.35, *p* = 0.002). Markar et al. [15] also analyzed 14,150 patients who underwent EVAR and 24,645 patients who underwent open repair. EVAR was associated with an increased risk in postoperative abdominal cancer (hazard ratio [HR], 1.14; 95% confidence interval [CI], 1.03–1.27) and all cancers (HR, 1.09; 95% CI, 1.02–1.17). In this rAAA cohort, where 17.2% of EVAR patients and 14.6% of OAR patients were diagnosed with cancer at five years (*p* = 0.328), no significant difference was found between the two operation methods.

Comparative data on rAAA patients are scarce in the literature, with only Troisi et al. [16] reporting on 405 patients who underwent endovascular and open rAAA repair, among whom 58.2% survived after 5 years. In this cohort, cancer was the most common cause of death among the 135 fatalities (n = 25; 18.5%).

Based on the present data, it can be cautiously concluded that the development of cancer adversely affects patient survival as early as three years after rAAA repair (Figure 3). Therefore, patients who have undergone rAAA repair should not be excluded from routine cancer screening. Given the smoking history of many of these patients, lung cancer screening in particular should be considered, as it is the most frequently observed tumor in this population.

The present study has several evident limitations. The comprehensiveness of the datasets relies on the coding accuracy of individual hospitals and the documentation provided by the health insurance company, which leaves room for potential coding errors. The data reflect the patient demographics of a specific health insurance company, capturing its social structure, and may not necessarily represent the entire German population. However, it is worth noting that AOK is the largest health insurance company in Germany, with a market share of 37%. The anonymity of the datasets prevented analysis of the treated hospitals and their case volumes. Additionally, the causes of death and the exact dates of death remained indeterminable. The aortic diameter is not included in the health insurance data. Conversely, a notable strength of this study lies in its ability to document the long-term survival of all patients for up to nine years.

## 5. Conclusions

In summary, the perioperative mortality rate for rAAA repair was lower with EVAR compared to OAR, but OAR was associated with better long-term survival. Women had less favorable survival outcomes than men, which can be attributed to their higher age. Even in octogenarians, rAAA repair is indicated, with survival rates of 19.9% after 5 years and even higher when perioperative deaths are excluded (38.4%). Cancer remains a significant risk factor for survival in patients with rAAA, with a survival rate after five years of 59.9% in cancer-free patients versus 50.5% in those who developed cancer. Given the high risk of lung cancer development, these patients should be closely monitored during follow-up.

## Figures and Tables

**Table 1:** Baseline characteristics of male and female patients undergoing ruptured abdominal aortic aneurysm repair (all patients were cancer-free at time of repair).

**Parameter**	**Male** **n = 2391**	**Female** **n = 542**	***p*** **-Value**
EVAR, n (%)	976 (40.8)	211 (38.9)	0.418
OAR, n (%)	1415 (59.2)	331 (61.1)	0.418
Age, mean ± SD in years, median (min–max)	73.9 ± 9.6, 75 (25–97)	79.6 ± 8.7, 81 (36–100)	<0.001
Patients ≥ 80 years, n (%)	719 (30.1)	314 (57.9)	<0.001
History of myocardial infarction, n (%)	201 (8.4)	28 (5.2)	0.011
History of stroke, n (%)	85 (3.6)	29 (5.4)	0.051
History of intracerebral bleeding, n (%)	9 (0.4)	1 (0.2)	0.700
History of TIA, n (%)	43 (1.8)	11 (2.0)	0.718
Arterial hypertension, n (%)	840 (35.1)	257 (47.4)	<0.001
Diabetes mellitus type 2, n (%)	258 (10.8)	86 (15.9)	0.001
COPD, n (%)	261 (10.9)	66 (12.2)	0.400
Left heart failure (NYHA 2–4 and unspecified), n (%)	220 (9.2)	159 (10.9)	0.227
Chronic kidney disease (stage 3–5), n (%)	201 (8.4)	56 (10.3)	0.152
-Stage 3, n (%)	−159 (6.6)	−45 (8.3)	0.172
-Stage 4, n (%)	−32 (1.3)	−10 (1.8)	0.370
-Stage 5, n (%)	−10 (0.4)	−1 (0.2)	0.701
PAD (Fontaine stage 3–4), n (%)	62 (2.6)	14 (2.6)	0.989
-Stage 3, n (%)	−26 (1.1)	−5 (0.9)	0.735
-Stage 4, n (%)	−36 (1.5)	−9 (1.7)	0.791

EVAR: endovascular aneurysm repair, OAR: open aneurysm repair, SD: standard deviation, min–max: minimum–maximum, TIA: transient ischemic attack, COPD: chronic obstructive pulmonary disease, NYHA: New York Heart Association, chronic kidney disease stage 3–5: glomerular filtration rate under 60 mL/min/1.73 m^2^, PAD: peripheral artery disease.

**Table 2:** Perioperative outcomes after ruptured abdominal aortic aneurysm repair.

**Parameter**	**Male** **n = 2391**	**Female** **n = 542**	***p*** **-Value**
Perioperative mortality, n (%)	770 (32.2)	225 (41.5)	<0.001
Perioperative mortality EVAR, n (%)	201/976 (20.6)	51/211 (24.2)	0.249
Perioperative mortality OAR, n (%)	569/1415 (40.2)	174/331 (52.6)	<0.001
Perioperative mortality < 80 years, n (%)	428/1627 (25.6)	67/228 (29.4)	0.222
Perioperative mortality ≥ 80 years, n (%)	342/719 (47.6)	158/314 (50.3)	0.416
LOS, mean ± SD in days, median (min-max)	20.7 ± 22.3, 14 (0–281)	21.6 ± 24.1, 15 (0–226)	0.802
LOS of surviving patients, mean ± SD in days, median (min–max)	26.6 ± 23.7, 18 (1–281)	30.3 ± 26.3, 23 (1–226)	0.002
Blood transfusions, n (%)	1842 (77.0)	452 (83.4)	0.001
Intensive care treatment, n (%)	1541 (64.5)	345 (63.7)	0.727
Wound complications, n (%)	235 (9.8)	45 (8.3)	0.275
Myocardial infarction, n (%)	118 (4.9)	19 (3.5)	0.154
Stroke, intracerebral bleeding or TIA, n (%)	58 (2.4)	18 (3.3)	0.236
Dialysis, n (%)	462 (19.3)	86 (15.9)	0.062
Pneumonia, n (%)	432 (18.1)	84 (15.5)	0.156
Deep-vein thrombosis, n (%)	28 (1.2)	5 (0.9)	0.620
Major amputation, n (%)	20 (0.8)	3 (0.6)	0.500
Ileus, n (%)	153 (6.4)	26 (4.8)	0.160

EVAR: endovascular aneurysm repair, OAR: open aneurysm repair, SD: standard deviation, min–max: minimum–maximum, TIA: transient ischemic attack, LOS: length of stay.

**Table 3:** Survival 5 years after ruptured abdominal aortic aneurysm repair (all patients were cancer-free at time of repair). All percentages are Kaplan–Meier estimates.

Survival After 5 Years	Male n = 2391	Female n = 542	*p*-Value
Total cohort, n (%)	1025/2391 (40.7)	171/542 (29.1)	<0.001
EVAR, n (%)	453/976 (42.7)	81/211 (34.9)	0.001
OAR, n (%)	572/1415 (39.2)	90/331 (27.1)	<0.001
Patients < 80 years, n (%)	868/1672 (49.8)	101/228 (41.4)	0.003
Patients ≥ 80 years, n (%)	157/719 (19.7)	70/314 (20.3)	0.867
EVAR (perioperative deaths excluded), n (%)	453/775 (53.8)	77/160 (41.8)	0.001
OAR (perioperative deaths excluded), n (%)	572/846 (65.5)	94/157 (57.1)	0.004
Patients < 80 years (perioperative deaths excluded), n (%)	868/1244 (67.0)	101/161 (58.7)	0.003
Patients ≥ 80 years (perioperative deaths excluded), n (%)	157/377 (37.5)	70/156 (40.9)	0.556

EVAR: endovascular aneurysm repair, OAR: open aneurysm repair.

**Table 5:** Cancer incidence 5 years after abdominal aortic aneurysm repair (all patients were cancer-free at time of repair and survived the intervention). All percentages are Kaplan–Meier estimates.

	Male n = 1621	Female n = 317	*p*-Value
Cancer incidence	16.5%	11.9%	0.153
Cancer incidence EVAR	18.2%	11.0%	0.272
Cancer incidence OAR	15.2%	12.1%	0.354
Cancer incidence < 80 years	15.1%	9.5%	0.251
Cancer incidence ≥ 80 years	22.7%	14.5%	0.139
Lung cancer incidence	4.4%	2.7%	0.288
Prostate cancer incidence	2.5%	0.0%	0.016
Colon cancer incidence	2.0%	1.4%	0.598
Ureter and bladder cancer incidence	2.8%	1.0%	0.098

EVAR: endovascular aneurysm repair, OAR: open aneurysm repair.

**Table 4:** Hazard ratio (HR) and proportional hazard model (multivariable analysis) for long-term mortality (all patients were cancer-free at time of repair). All patients included in the analysis survived the initial intervention.

	HR	95% CI	*p*-Value
Age ≥ 80 years	2.013	1.747–2.321	<0.001
COPD	1.602	1.303–1.970	<0.001
Chronic kidney disease (stage 3–5)	1.545	1.232–1.937	<0.001
Cancer	1.318	1.112–1.564	0.002
Left heart failure (NYHA 2–4 and unspecified)	1.269	1.008–1.599	0.043
OAR (vs. EVAR)	0.838	0.731–0.960	0.011
Women (vs. Men)	1.161	0.980–1.376	0.084
History of myocardial infarction	1.170	0.915–1.497	0.211
History of stroke, intracerebral bleeding, or TIA	1.050	0.779–1.415	0.749
Arterial hypertension	1.076	0.919–1.261	0.362
Diabetes mellitus type 2	1.112	0.898–1.377	0.331
PAD (Fontaine stage 3–4)	1.091	0.727–1.638	0.673

EVAR: endovascular aneurysm repair, OAR: open aneurysm repair, HR: hazard ratio, CI: confidence interval, TIA: transient ischemic attack, COPD: chronic obstructive pulmonary disease, chronic kidney disease stage 3–5: glomerular filtration rate under 60 mL/min/1.73 m^2^, NYHA: New York Heart Association, PAD: peripheral artery disease.

## Data Availability

The collected datasets can be requested in anonymized form from the corresponding author upon justified request.
